# Indonesia's Domestic Biogas Programme – Household panel survey data

**DOI:** 10.1016/j.dib.2018.02.083

**Published:** 2018-03-07

**Authors:** Arjun S. Bedi, Robert Sparrow, Luca Tasciotti

**Affiliations:** a International Institute of Social Studies, Erasmus University Rotterdam, Kortenaerkade 12, 2518 AX, The Hague, The Netherlands; b Wageningen University, Hollandseweg 1, 6706 KN Wageningen, The Netherlands; c School of Oriental and African Studies, University of London, 10 Thornhaugh Street, Russell Square, London WC1H 0XG, UK

## Abstract

The data presented in this article are related to the research paper titled, “The impact of a household biogas programme on energy use and expenditure in East Java” (A.S. Bedi, R. Sparrow, L. Tasciotti, 2017) [1]. This Data in Brief article presents two rounds of survey data conducted in 2011 and 2012 for a panel of 677 dairy farm households in the province of East Java, Indonesia. The survey relied on structured questionnaires to collect data on the production and use of biogas, the use of other non-renewable energy sources, farm characteristics, and socioeconomic and demographic characteristics of households. The panel data set in STATA format and do files are made publicly available to promote replicability and extended analyses of a sustainable energy initiative.

**Specifications Table**

**Table:** 

Subject area	*Economics*
More specific subject area	*Energy economics, development economics, agricultural economics*
Type of data	*STATA data set and do files*
How data was acquired	*Survey*
Data format	*Filtered and analyzed*
Experimental factors	*Survey data of dairy farm households in East Java*
Experimental features	*Farm household energy use, biogas production, farm characteristics, socioeconomic characteristics*
Data source location	*East Java, Indonesia*
Data accessibility	*Data is with this article*

**Value of the data**

• Unique data of energy use and biogas production for a panel of dairy farm households in East Java, Indonesia.
• Displays how access to biogas alters household use of renewable and non-renewable energy sources for cooking.
• Allows an assessment of the various benefits obtained from using biogas.
• Permits cost-benefit calculations associated with investing in biogas producing units.
• The data set and do files will enable other researchers to replicate the current study and to carry out extended analyses of a sustainable energy initiative.

## 1 Data

Two survey rounds of the same dairy farm households were conducted in May-June 2011 and May-June 2012 in East Java province, Indonesia. The surveys were conducted in East Java as this province has the bulk of the biogas digesters installed through the Indonesia Domestic Biogas Programme (BIRU). Prior to carrying out the survey, informed consent was obtained from all survey participants.

## 2 Experimental Design, Materials and Methods

The sampling frame consists of dairy farmers participating in the cooperatives that are involved as Construction Partner Organization (CPO) in the BIRU programme. Of the 11 CPOs in East Java, 9 were selected for the survey while 2 had too few installed biogas digesters to be included. Sampling of dairy farms was stratified by CPO and by BIRU participation status (users of biogas digesters, applicants for a digester and non-applicants).

The total sampling pool included 2,086 current users, 497 applicants and 18,321 non-applicants who satisfied basic BIRU eligibility criteria (own at least one productive cow, regularly supply milk to the cooperative and not own a digester through another program). The number of households randomly sampled for the survey in 2011 is shown in Table 1 - in total 695 households of which 101 bio-digester users, 250 applicants and 344 non-applicants. The distribution of sampled applicants across CPOs was proportional to the relative share of the applicants in each CPO, while the distribution of sampled non-applicants and existing users was proportional to the underlying distribution of the CPO populations.

**Table 1:** Number of farmers sampled from the three groups in 2011, by CPO.

CPO	Current users	Applicants	Non-applicants	Total
Kan Jabung	8	4	5	17
LPKP	9	23	32	64
Sumber Makmur Ngantang	14	21	29	64
KPUB Sapi Jaya	2	15	21	38
Sami Mandiri	12	14	20	46
Sae Pujon	21	65	87	173
Kud Dadi Jaya	5	13	18	36
Setia Kawan	22	86	119	227
Kud Semen	8	9	13	30
Total	101	250	344	695

For the second survey round, in 2012, the same households were interviewed. The attrition rate was 2.6 percent, as 677 out of 695 households were located for follow-up survey. We found no evidence of systematic differences between households in the panel and those who dropped out.

The household questionnaire included modules on a wide-range of socio-economic characteristics, farm characteristics, cooking behavior, energy use, energy related expenses, and a detailed module on bio-gas digesters.

