# Supplementary material for: Indonesia's Domestic Biogas Programme – Household panel survey data
Source: Data Brief. 2018 Mar 7;17:1388–90. doi: 10.1016/j.dib.2018.02.083 (PMC5854924; doi:10.1016/j.dib.2018.02.083)
Supplement: Supplementary file 1 — Supplementary material [file mmc1.docx]

Conflict of interest

There is no conflict of interest.
